# Supplementary material for: A whole-animal phenotypic drug screen identifies suppressors of atherogenic lipoproteins
Source: eLife. 2026 Aug 27;14:RP105314. doi: 10.7554/eLife.105314 (PMC13521566; doi:10.7554/eLife.105314)
Supplement: Supplementary file 2 [file elife-105314-code1.zip › Output_data/BIM5078_nPAGE.pdf]

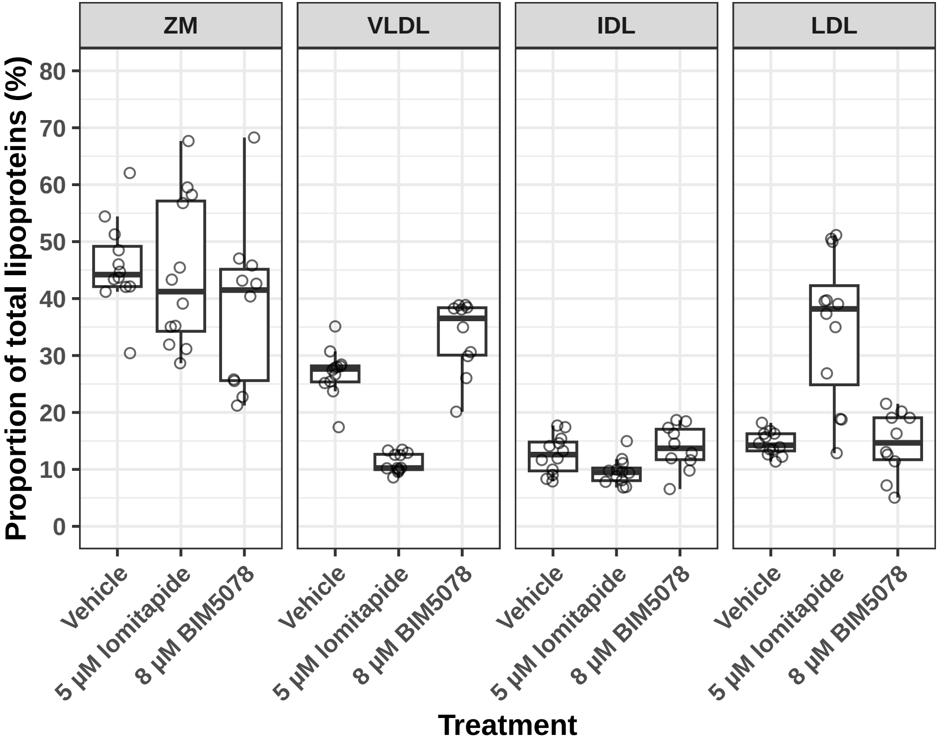
ZM
VLDL
IDL
LDL
Proportion of total lipoproteins (%)
0
10
20
30
40
50
60
70
80
Vehicle
5 µM lomitapide
8 µM BIM5078
Treatment
